# Supplementary material for: Polyketide synthase gene clusters in the ericoid mycorrhizal fungus Oidiodendron maius: genome-wide prediction, expression profiling, and functional characterization of knockout mutants
Source: Front Plant Sci. 2026 Sep 16;17:1924430. doi: 10.3389/fpls.2026.1924430 (PMC13623903; doi:10.3389/fpls.2026.1924430)
Supplement: Supplementary file 1 [file SupplementaryFile1.zip › Supplementary Materials/Supplementary Folder 1.zip!/Oidma1_AssemblyScaffolds_Repeatmasked/knownclusterblast/cluster47/jgi.p_Oidma1_106799_mibig_hits.html]

| MIBiG Protein | Description | MIBiG Cluster | MiBiG Product | % ID | % Coverage | BLAST Score | E-value |
| --- | --- | --- | --- | --- | --- | --- | --- |
| EFY94489.1 | laccase\_Lcc4 | BGC0000337 | Destruxins\_biosynthetic\_gene\_cluster | 38 | 97.3244147157 | 379 | 9e-105 |
| EAL89346.1 | extracellular\_dihydrogeodin\_oxidase/laccase,\_putative | BGC0001403 | Trypacidin\_biosynthetic\_gene\_cluster | 36 | 102.006688963 | 347 | 2.9e-95 |
| AGO59052.1 | PtaK | BGC0000121 | Pestheic\_acid\_biosynthetic\_gene\_cluster | 36 | 89.7993311037 | 289 | 1.2e-77 |
| AGO59042.1 | PtaE | BGC0000121 | Pestheic\_acid\_biosynthetic\_gene\_cluster | 34 | 89.7993311037 | 256 | 6.8e-68 |
